# Prognostic value of carcinoembryonic antigen in colorectal adenocarcinoma: expanding hypotheses into clinical practice

**DOI:** 10.1007/s10238-024-01547-1

**Published:** 2025-01-03

**Authors:** Adam Thomas Cristaudo, David Lewis Morris

**Affiliations:** Liver & Peritonectomy Unit, Department of Surgery, St George Hospital, Pitney Building, Short Street, Kogarah, NSW 2217 Australia

## Abstract

**Purpose:**

This study seeks to resolve a fundamental question in oncology: Why do appendiceal and colorectal adenocarcinomas exhibit distinct liver metastasis rates? Building on our prior hypothesis published in the *British Journal of Surgery*, our institution has investigated potential DNA mutations within the carcinoembryonic antigen-related cell adhesion molecule (CEACAM5) gene’s Pro-Glu-Leu-Pro-Lys (PELPK) motif to evaluate its role as a biomarker for liver metastasis risk.

**Methods:**

Partnering with the Australian Genome Research Facility, the PELPK motif of CEACAM5 was analysed in colorectal and appendiceal adenocarcinomas to detect DNA mutations associated with liver metastasis. Additionally, our institution performed the COPPER trial to assess carcinoembryonic antigen (CEA) levels in portal versus peripheral blood in patients with appendiceal adenocarcinoma and a systematic review and meta-analysis of 136 studies on CEA’s prognostic significance among patients with colorectal and appendiceal adenocarcinoma.

**Results:**

No mutations were identified within the PELPK region. The COPPER trial further demonstrated no statistically significant differences in CEA levels between portal and peripheral blood in appendiceal adenocarcinoma. However, the systematic review and meta-analysis confirmed CEA’s prognostic role in patients with appendiceal or colorectal adenocarcinoma.

**Conclusion:**

The absence of DNA mutations suggests that metastatic potential may be driven by downstream mRNA or protein modifications in the CEA PELPK region. Future work will include surface plasmon resonance studies to investigate CEA–receptor interactions and development of immunohistochemistry for CEA PELPK expression. Such findings are poised to redefine global strategies for cancer stratification and targeted immunotherapy, setting the stage for groundbreaking advancements in cancer prognosis and patient outcomes.

Carcinoembryonic antigen (CEA) has been widely recognized as a biomarker for colorectal cancer, specifically its potential role in surveillance and in predicting liver metastasis. In 2022, our first author published a hypothesis in the *British Journal of Surgery* to address the observed discrepancy in liver metastasis rates between patients with appendiceal versus colorectal adenocarcinoma [1]. This correspondence expands on that hypothesis, presenting two novel findings: the genetic stability of the Pro-Glu-Leu-Pro-Lys (PELPK) region in the carcinoembryonic antigen-related cell adhesion molecule (CEACAM) 5 gene and a partial validation of our original hypothesis [2, 3].

In collaboration with the Australian Genome Research Facility, our institution investigated the PELPK motif within the CEA molecule to identify any DNA mutations that might correlate with a higher risk of liver metastasis in patients with colorectal or appendiceal adenocarcinoma. No mutations were detected in this motif, underscoring CEA’s reliability as a prognostic marker across both cancer types. This absence of DNA mutations suggests that metastatic drivers may act downstream, likely at the mRNA or protein level. Further investigation of these pathways could reveal modifications linked to a higher risk of metachronous liver metastasis, highlighting the need for research on post-transcriptional and post-translational changes in the CEA PELPK region.

The recently completed COPPER trial—our institution’s investigation into peripheral versus portal CEA blood levels in patients with appendiceal adenocarcinoma—revealed no significant difference between these sampling sites [4]. Additionally, our systematic review and meta-analysis of 136 studies (1972–2021) confirmed CEA’s prognostic significance, demonstrating elevated CEA levels in appendiceal adenocarcinoma patients compared to those with colorectal adenocarcinoma (MD: 14%; *p* = 0.0001) [5]. These findings corroborate those of Tabuchi et al. [6] and support including high-risk patients in the forthcoming CCaLM trial [7].

Looking toward the future, our institution is focusing on surface plasmon resonance experiments to explore the binding affinity between CEA and its receptor, CEAR. This research aims to clarify the molecular interactions that may drive metastatic behavior and examine the potential of monoclonal antibodies (MAbs) to target these interactions. Additionally, we plan to develop an immunohistochemistry test for the CEA PELPK motif to better stratify patients at risk for metachronous liver metastasis. This stratification will facilitate a randomized control trial of MAbs with the potential to improve five-year survival rates by 40 to 60 percent in high-risk colon cancer patients within the CCaLM trial framework.

Our institution is confident that this work will advance both the prognostic and therapeutic applications of CEA and its PELPK motif, forging a path toward more targeted treatment options for colon cancer patients worldwide.

## Data Availability

No datasets were generated or analysed during the current study.
